# Combined Application of Bacteriophages and Carvacrol in the Control of *Pseudomonas syringae* pv. *actinidiae* Planktonic and Biofilm Forms

**DOI:** 10.3390/microorganisms8060837

**Published:** 2020-06-02

**Authors:** Peien Ni, Lei Wang, Bohan Deng, Songtao Jiu, Chao Ma, Caixi Zhang, Adelaide Almeida, Dapeng Wang, Wenping Xu, Shiping Wang

**Affiliations:** 1 School of Agriculture and Biology, Shanghai Jiao Tong University, Shanghai 200240, China; wayneni11@hotmail.com (P.N.); leiwang2016@sjtu.edu.cn (L.W.); bohandeng@163.com (B.D.); jiusongtao@sjtu.edu.cn (S.J.); chaoma2015@sjtu.edu.cn (C.M.); acaizh@sjtu.edu.cn (C.Z.); norovirus@163.com (D.W.); 2 Department of Biology and CESAM, University of Aveiro, Campus Universitário de Santiago, 3810-193 Aveiro, Portugal; aalmeida@ua.pt

**Keywords:** bacteriophage, carvacrol, inhibit, *Pseudomonas syringae* pv. *actinidiae*

## Abstract

*Pseudomonas syringae* pv. *actinidiae* (Psa) is the causative agent of the bacterial canker of kiwifruit (*Actinidia* spp.). Phage therapy has been suggested as a viable alternative approach to controlling this disease, but its efficacy is limited by the emergence of phage-resistant mutants. Carvacrol is an essential oil that may be useful for the control of Psa. Combination therapies can be used to overcome resistance development. Here, the combination of phages (single phage suspensions of phages PN05 and PN09, and a cocktail of both phages) and carvacrol was investigated in controlling Psa planktonic and biofilm forms in vitro. The phage therapy alone (with phages PN05 and PN09), and the carvacrol alone (minimum inhibitory concentration 2.0 mg/mL), inhibited Psa growth, but the combined effect of both therapies was more effective. The phages alone effectively inhibited Psa growth for 24 h, but Psa regrowth was observed after this time. The carvacrol (2.0 mg/mL) alone prevented the biofilm formation for 48 h, but did not destroy the pre-formed biofilms. The combined treatment, phages and carvacrol (2.0 mg/mL), showed a higher efficacy, preventing Psa regrowth for more than 40 h. In conclusion, the combined treatment with phages and carvacrol may be a promising, environment-friendly and cost-effective approach to controlling Psa in the kiwifruit industry.

## 1. Introduction

*Pseudomonas syringae* pv. *actinidiae* (Psa) is an important phytopathogen that causes the bacterial bleeding canker disease in kiwifruit (*Actinidia* spp.) [1], affecting the kiwifruit industry worldwide. The disease manifests as brown leaf spots with chlorotic haloes, fruit specks and scabs, brown discoloration of buds, and cankers with exudates on trunks and twigs [2]. Like other *Pseudomonads*, Psa can form biofilms, which protect Psa from host defense and bactericidal compounds [3].

Conventional methods for controlling bacterial bleeding canker in kiwifruit, including copper derivatives and antibiotics, have their limitations. Further, the massive use of copper and antibiotics can lead to the emergence of resistance in the pathogen, changes in bacterial communities, and pollution of the environment [4]. Phages offer a promising alternative for Psa control. Phages are bacterial viruses that exhibit high host specificity and strong lytic activity [5]. In our previous study, three lytic phages of Psa were isolated and characterized [6]. Other studies also described new phages active against Psa [7,8], but only a few studies have evaluated the therapeutic potential of phages in controlling this phytopathogen [9,10]. No studies with phage cocktails, or with combined therapies including phages, have yet been evaluated against Psa; approaches that, besides increasing the effectiveness of the treatment, prevent the development of bacterial resistance to phages [11,12,13,14].

Similar to phages, plant essential oils can be used as an alternative natural antibacterial approach to the conventional treatments with antibiotics. Certain essential oils can inhibit the growth of bacteria, yeasts, and molds [15]. Among them, carvacrol, also known as 5-isopropyl-2-methylphenol, is an oxygenated monoterpenoid [16], with a broad-spectrum of antibacterial activity against pathogens such as *Escherichia coli* [17], *Staphylococcus aureus* [18] and *Listeria monocytogenes* [19], and which thus represents a promising natural antibacterial compound. The carvacrol’s antibacterial mechanisms include disruption of the cell membrane, induction of reactive oxygen species production, and inhibition of efflux pumps, biofilm formation and quorum sensing [20,21,22,23,24,25]. However, to our knowledge, few attempts have been made to control Psa using a combination of phages and carvacrol [26,27].

This study aimed to evaluate the effects of the combined treatment, with Psa-specific phages and carvacrol, on Psa, and in particular, on biofilm formation and destruction of the pre-formed biofilm.

## 2. Materials and Methods

### 2.1. Bacterial Strains and Culture Conditions

The three strains of Psa (SCJY02-1, 4LH1402-1, JH1401-1) used in this study were described in our previous report [6]. They belong to biovar 3, which comprises the most virulent epidemic pathogenic strain identified in the last decade. The Psa strains were cultured in nutrient broth (NB; HuanKai Microbial, Guangzhou, China) at 27 °C under shaking at 150 rpm. SCJY02-1 was used as the only test strain in all experiments, except in the determination of the minimum inhibitory concentration (MIC) of carvacrol against Psa, where all three strains were used.

### 2.2. Preparation of Phages

Phages (PN05 and PN09) were isolated from water samples collected in Hangzhou, China. SCJY02-1 strain was used as the host for phage isolation and propagation. PN05 and PN09 are double-stranded DNA phages that belong to the family *Myoviridae*. Phages were stored at 4 °C. Briefly, for phage propagation, 200.0 μL of log-phase Psa culture (approximately 10^9^ CFU/mL) was combined with 100.0 μL of phages in 15.0 mL of 0.7% NB (soft agar). After overnight incubation at 27 °C, the phages were recovered by adding 5.0 mL of SM buffer (10 mM Tris-HCl, pH 7.5; 100 mM NaCl; 10.0 mM MgSO_4_; 0.01% gelatin) on top of the plates. The plates were kept at 25 °C for 4 h under shaking. Then, the agar and liquid were scraped off the plate and centrifuged at 8000 *g* for 10 min. Chloroform (50.0 μL) was added to the supernatant (5.0 mL), which was then stored at 4 °C until use. Each individual phage solution was prepared at a concentration of approximately 1.0 × 10^10^ PFU/mL. A phage cocktail was prepared by combining equal volumes of each phage solution, and the concentration of phage cocktail was approximately 1.0 × 10^10^ PFU/mL.

### 2.3. Preparation of Carvacrol

Carvacrol (99% pure) was purchased from MACKLIN (Shanghai, China). A 100.0 mg/mL stock solution of carvacrol was prepared in 100% ethanol. A working solution (4.0 mg/mL) of carvacrol was prepared by diluting the stock solution in NB.

### 2.4. Evaluation of Phage Sensitivity to Carvacrol

To test the phage sensitivity to carvacrol, the phages (approximately 10^9^ PFU/mL) were exposed to different concentrations of carvacrol (0.5 mg/mL, 1.0 mg/mL, 2.0 mg/mL and 4.0 mg/mL) at 4 °C for 24 h. Ethanol at 2% without carvacrol was used as the negative control. The phage titer was determined by the double-layer agar plate method [6].

### 2.5. Evaluation of Phage Bacteriolytic Activity In Vitro

To evaluate the phage bacteriolytic activity, overnight cultures of Psa (SCJY02-1) were diluted (1:100) in fresh NB and incubated at 27 °C under shaking at 150 rpm until the log-phase (approximately 10^9^ CFU/mL) was reached. Phage stock dilutions were then added at multiplicities of infection (MOIs) of 0.1, 1, 10 and 100. Psa cultured in SM buffer was used as control. After the addition of different treatments, bacteriolytic activity was monitored by measuring the optical density at 600 nm (OD_600_) at 1-h intervals for 40 h. For each condition three independent assays were done, with two technical replicates per assay.

### 2.6. Determination of Antimicrobial Activity of Carvacrol

The minimum inhibitory concentrations (MIC) of carvacrol against Psa strains SCJY02-1, 4LH1402-1, JH1401-1 were determined using a standard protocol described by the Clinical and Laboratory Standards Institute [28]. The assay was conducted using 96-well plates. The final concentrations of carvacrol ranged from 0.5 mg/mL to 2.0 mg/mL in NB. Ethanol at 2%, which did not inhibit the growth of the Psa strains evaluated, was used as the negative control. The MIC was identified as the lowest concentration of carvacrol resulting in no visible growth of bacterial cells after incubation at 27 °C for 48 h. Psa growth during exposure to carvacrol was monitored by measuring the OD_600_.

To evaluate the susceptibility of Psa strain SCJY02-1 to carvacrol, the bacteria were incubated with various concentrations of carvacrol (0.5 mg/mL, 1.0 mg/mL and 2.0 mg/mL in NB) at 27 °C and growth was monitored by measuring the OD_600_ after the addition of different treatments every hour for 40 h. The assay was conducted using 96-well plates. A quantity of 500 μL of an overnight culture was diluted in 25.0 mL of NB to achieve an OD_600_ of 0.1 μL, and 100.0 μL of the cell suspension and 100.0 μL of carvacrol solution were added to each well. Wells without carvacrol were used as a negative control. For each condition three independent assays were done, with two technical replicates per each assay.

### 2.7. Evaluation of the Efficacy of Combined Phages and Carvacrol against Psa

The susceptibility of Psa strain SCJY02-1 to combinations of the phages and carvacrol at different concentrations was evaluated by measuring Psa growth (OD_600_) after the addition of different treatments at 27 °C every hour for 40 h. We evaluated each single phage and the cocktail in combination with 0 mg/mL, 0.5 mg/mL, 1.0 mg/mL or 2.0 mg/mL carvacrol. Psa without phage added was used as a control. Phage combined with varying concentrations of carvacrol was added to Psa inoculum at a MOI of 1 for experimental setups.

To determine the inactivation of Psa strain SCJY02-1 by the phages and carvacrol, a Psa culture (final concentration of 10^9^ CFU/mL) was mixed with phages (final concentration of 1 × 10^9^ PFU/mL) and carvacrol (final concentration of 2.0 mg/mL) and incubated at 27 °C. Samples were collected at 0 h, 4 h, 8 h, 12 h, 16 h, 20 h, 24 h, 28 h, 32 h, 36 h and 40 h of incubation, and the bacterial concentration after incubation in NA at 27 °C for 48 h was determined by the colony count method. According to the results presented in Section 3.4, PN05 was more effective than PN09 at inhibiting Psa growth. Therefore, we evaluated PN05 or phage cocktail in combination with 2.0 mg/mL carvacrol. The following five treatments were used: (1) PN05, (2) phage cocktail, (3) carvacrol alone, (4) PN05 + carvacrol, and (5) phage cocktail + carvacrol. Psa culture without phage and carvacrol added was used as a control. For each condition three independent assays were done, with two technical replicates per assay.

### 2.8. Biofilm Inhibition Assay

The ability of the phage and carvacrol to inhibit biofilm formation by Psa was evaluated using a plate staining assay as previously reported [29], with minor modifications. The following five treatments were used: (1) phage alone (1 × 10^9^ PFU/mL), (2) phage cocktail (1 × 10^9^ PFU/mL), (3) carvacrol alone (2.0 mg/mL), (4) phage (1 × 10^9^ PFU/mL) + carvacrol (2.0 mg/mL), and (5) phage cocktail (1 × 10^9^ PFU/mL) + carvacrol (2.0 mg/mL). The different treatment solutions (100.0 μL) were mixed with 200.0 μL of Psa culture (OD_600_ = 0.1) in 96-well plates. As a control, 100.0 μL of SM buffer with 200.0 μL Psa culture was added to the plates. The plates were incubated at 27 °C for 12 h, 24 h, 36 h or 48 h to form biofilm. Then, the medium was removed and each well was washed three times with 300.0 μL of phosphate-buffered saline (PBS). After removing the washing buffer, the plates were placed in an oven at 60 °C (Yi Heng Scientific Instruments, Shanghai, China) and dried for 1 h. Then, for biofilm staining, 300.0 μL of 0.1% crystal violet solution was added in each well and the plate was incubated at room temperature for 15 min. After gently washing with PBS, the stained biofilm was dried. Glacial acetic acid (30%, 300.0 μL) was added to dissolve the stain. The OD_600_ was measured using a Sunrise-Basic instrument (Tecan, Grodig, Austria). For each condition three independent assays were done, with two technical replicates per assay.

### 2.9. Pre-Formed Biofilm Destruction Assay

The ability of the phage and carvacrol to eliminate pre-formed biofilm was evaluated as described above, with the following adjustments. Psa culture (200.0 μL) was placed in each well of 96-well plates and the plates were incubated at 27 °C for 36 h (based on results presented in Section 3.5). Then, the supernate was removed and each well was washed three times with 200.0 μL of PBS. One of the plates was used to measure biofilm formation by staining with crystal violet. The other plates were used to evaluate the effects of the combined phage and carvacrol treatments described in Section 2.8. The different treatment solutions (200 μL) were added to the wells, and the plates were incubated at 27 °C for 24 h to allow biofilm destruction. As a control, 200.0 μL SM buffer was used. The medium was removed and biofilms were stained as described above. The OD_600_ was measured using the Sunrise-Basic instrument. For each condition three independent assays were done, with two technical replicates per assay.

### 2.10. Statistical Analysis

Statistical Analysis Software (SAS, version 9.2, Raleigh, NC, USA) was used for statistical analysis. Data were analyzed by ANOVA and the least significant difference method. Differences were considered significant at *p* < 0.05.

## 3. Results

### 3.1. Phage Sensitivity to Carvacrol

**Figure 1:**
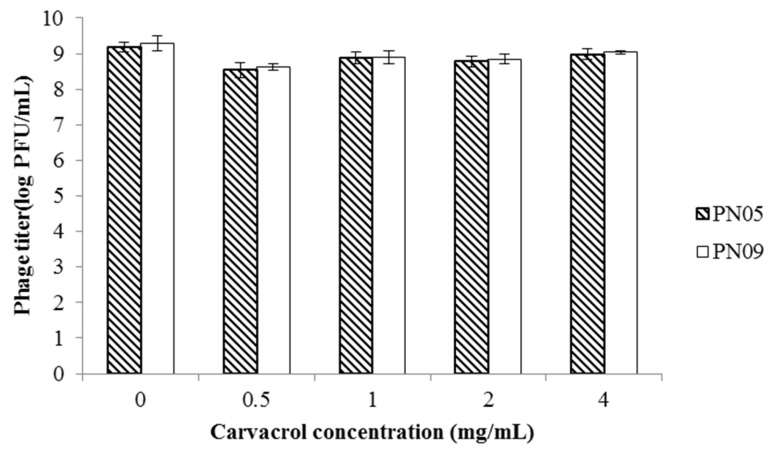
Phage sensitivity to carvacrol. The sensitivity of the phages to the indicated concentrations of carvacrol was evaluated after a 24-h incubation at 4 °C. Data are the mean ± standard deviation of three independent assays.

The viability of both PN05 and PN09 was hardly affected after a 24-h incubation, at 4 °C and in the presence of 0.5 mg/mL, 1.0 mg/mL, 2.0 mg/mL or 4.0 mg/mL carvacrol (Figure 1).

### 3.2. Phage Bacteriolytic Activity In Vitro

**Figure 2:**
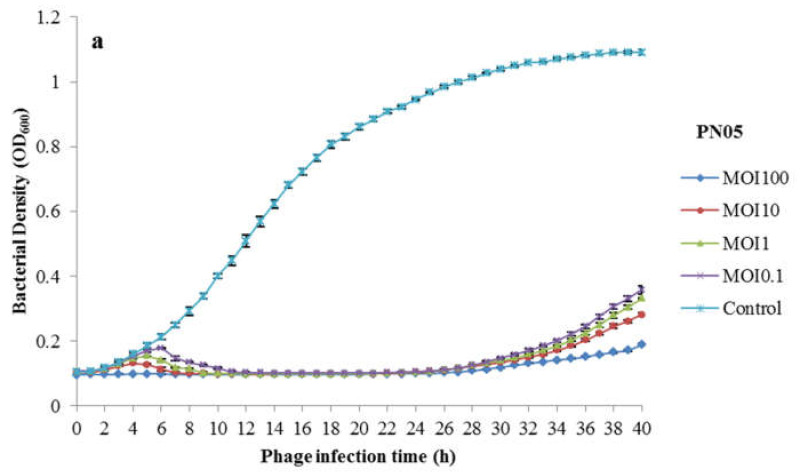

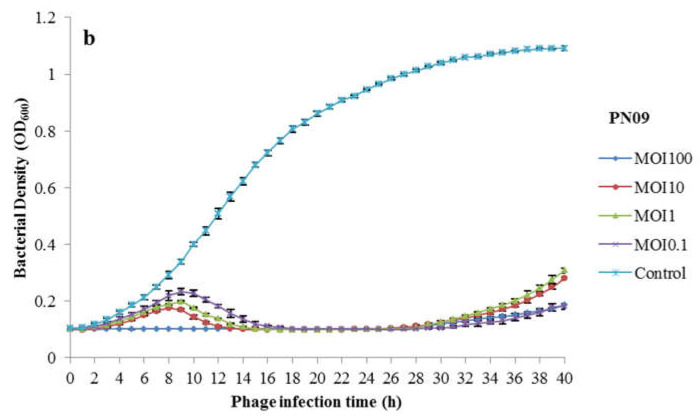
Bacteriolytic activity of the phages against Psa (SCJY02-1) in vitro. Early exponential cultures of Psa were treated with (**a**) PN05 or (**b**) PN09 at MOIs of 0.1, 1, 10 and 100, respectively. Psa culture inoculated with SM buffer instead of phage diluent was used as a negative control. Data are the mean ± standard deviation of three independent assays.

The bacteriolytic activity of PN05 and PN09 was assessed by inoculating the phages with a Psa strain (SCJY02-1) in culture broth, and monitoring bacterial growth. As shown in Figure 2a, Psa was completely inhibited for 12 h when treated with PN05 at different MOIs (0.1, 1 and 10), and no growth was observed until 22 h. However, the OD_600_ started to increase after 22 h. At MOI = 100, Psa was completely inhibited for up to 24 h, and then some regrowth was observed. As shown in Figure 2b, Psa was completely inhibited for 18 h when treated with PN09 at different MOIs of 0.1, 1 and 10, and no growth was observed until 26 h, but the OD_600_ started to increase after 26 h. At MOI = 100, Psa was completely inhibited for up to 26 h, and regrowth was observed after this time.

### 3.3. Antimicrobial Activity of Carvacrol

**Figure 3:**
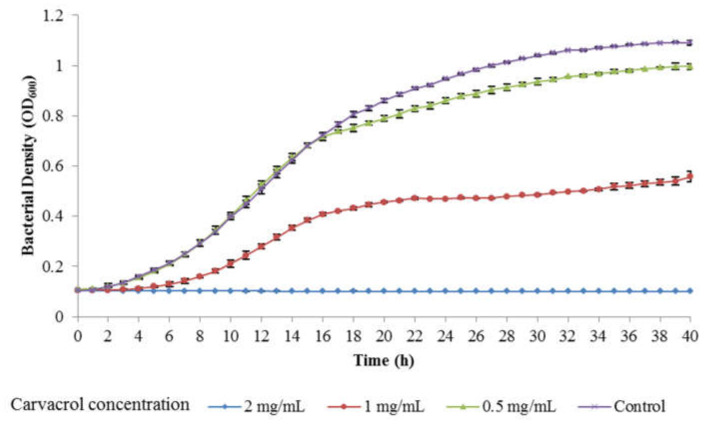
Growth inhibition of Psa (SCJY02-1) by carvacrol at different concentrations (0.5 mg/mL, 1 mg/mL and 2 mg/mL). Psa without carvacrol was used as control. Data are the mean ± standard deviation of three independent assays.

The MICs of carvacrol against Psa strains SCJY02-1, 4LH1402-1 and JH1401-1 in NB broth were 2.0 mg/mL. The inhibitory effect of carvacrol at different concentrations was also investigated by growth curve analysis. As shown in Figure 3, Psa growth in NB broth was completely inhibited by 2.0 mg/mL carvacrol, whereas it was only slightly inhibited by 0.5 mg/mL. This result was in line with that of the MIC determination.

### 3.4. Efficacy of Combined Phage and Carvacrol against Psa

The inhibitory effect of phages combined with carvacrol was evaluated by measuring the growth of the Psa strain SCJY02-1 in the presence of carvacrol at three concentrations (0.5 mg/mL, 1.0 mg/mL, or 2.0 mg/mL), as well as phages (single suspensions or cocktail, MOI = 1), at 27 °C for 40 h. The phage sensitivity assay described in Section 3.1 confirmed that carvacrol did not inactivate PN05 and PN09 at the concentrations used in this study.

**Figure 4:**
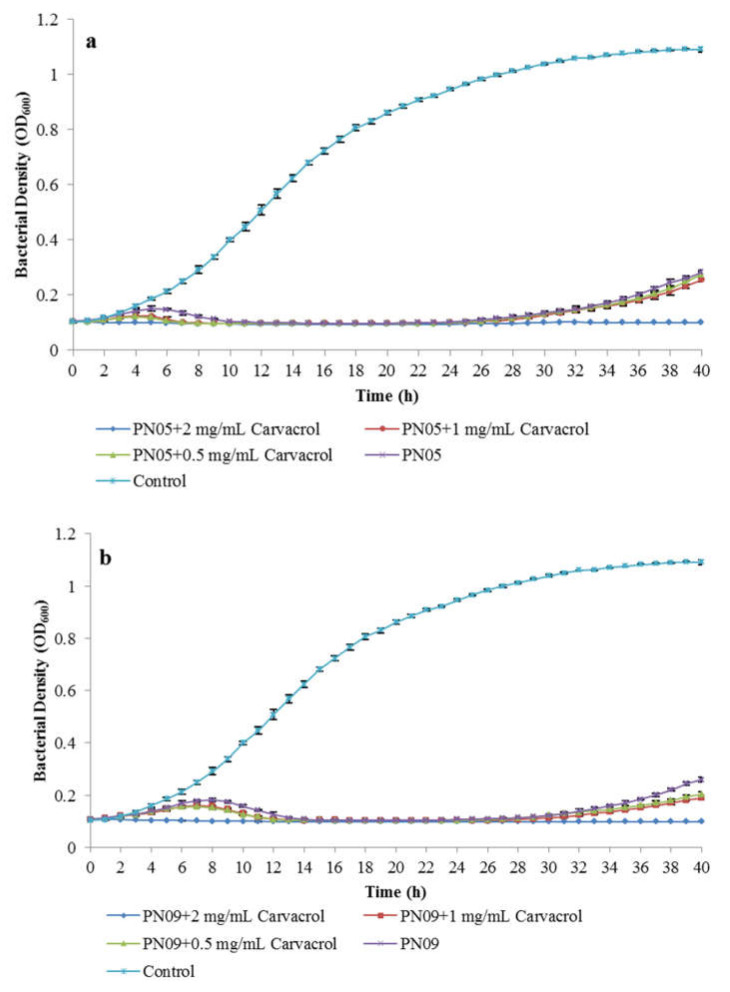

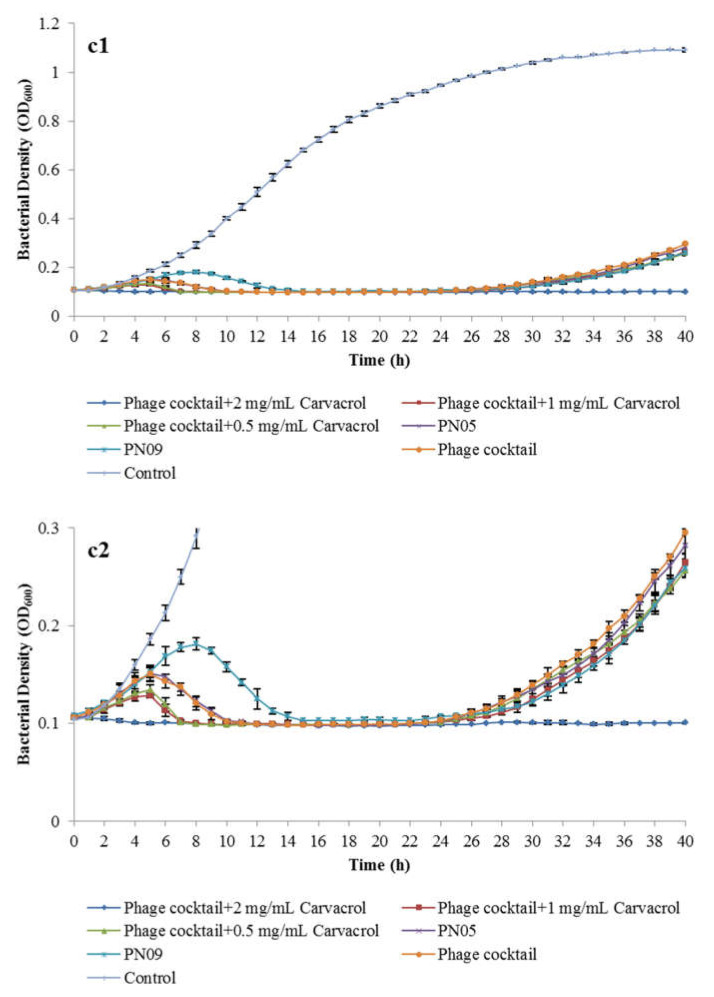
Psa (SCJY02-1) growth inhibition assay of phages (MOI = 1) and carvacrol. (**a**) PN05 and carvacrol, (**b**) PN09 and carvacrol, (**c1**,**c2**) phage cocktail and carvacrol (c2 is an enlarged image of c1) were used. Psa culture without phage or carvacrol was used as the control. Data are the mean ± standard deviation of three independent assays.

Psa incubated with phages exhibited a significant reduction in growth when compared to the non-treated control (Figure 4). In Figure 4a,b, PN05 or PN09 combined with 2.0 mg/mL carvacrol was the most efficient in inhibiting Psa growth, which was completely inhibited for up to 40 h. As shown in Figure 4a, PN05 + carvacrol (1.0 mg/mL) was more efficient than the phage alone. The bacterial density of Psa reached a maximum of approximately 0.120 (OD_600_) after 4 h, and Psa was completely inhibited after 8 h, whereas in the presence of PN05 alone, the maximum OD_600_ was 0.150 after 5 h and Psa was completely inhibited only at 12 h. The efficiency of PN05 + carvacrol (0.5 mg/mL) was similar to that of PN05 + carvacrol (1.0 mg/mL). As shown in Figure 4b, PN09 + carvacrol (1.0 mg/mL) inhibited Psa growth more effectively than phage alone; the Psa density reached a maximum OD_600_ of 0.160 after 7 h, and Psa was completely inhibited after 13 h, whereas in the presence of PN09 alone, the maximum OD_600_ was 0.180 after 8 h and Psa was completely inhibited after 15 h. PN09 + carvacrol (0.5 mg/mL) was as efficient as PN09 + carvacrol (1.0 mg/mL). However, Psa regrowth was observed in all treatments, except for phages + carvacrol (2.0 mg/mL) (Figure 4a,b). As shown in Figure 4c, PN09 alone showed the lowest efficiency in inhibiting Psa growth, as the maximum bacterial density of Psa here (OD_600_ = 0.180) was significantly higher (*p* < 0.05) than those recorded for the other treatments, and inhibition to the detection limit of the method was reached later than in the other treatments. The phage cocktail and PN05 alone showed similar efficiency in inhibiting Psa growth, as there was no significant difference (*p* > 0.05) between their maximum bacterial densities of Psa (OD_600_ = 0.151 and 0.150, respectively), and Psa was completely inhibited after 12 h. When the phage cocktail was combined with carvacrol at 0.5 mg/mL, 1.0 mg/mL or 2.0 mg/mL, the inhibitory effect was greater than that of the phage cocktail or each phage alone (Figure 4c). Psa was completely inhibited at an earlier time point when treated with the phage cocktail + carvacrol than when treated with the phage cocktail or each phage alone. However, Psa regrowth was also observed when the concentration of carvacrol was lower than 2.0 mg/mL. When compared with single phage + carvacrol, phage cocktail + carvacrol (2.0 mg/mL) showed effectivity similar to that of the single phage + carvacrol (2.0 mg/mL), and Psa was completely inhibited within 40 h. When Psa was treated with phage cocktail + carvacrol (1.0 mg/mL), the bacterial density reached a maximum of approximately 0.128 (OD_600_) after 5 h, and Psa was completely inhibited after 8 h, whereas in the presence of PN05 + carvacrol (1.0 mg/mL), the maximum OD_600_ was 0.120 after 4 h and Psa was completely inhibited at 8 h. There was a significant difference between the maximum bacterial density for phage cocktail + carvacrol (1.0 mg/mL) and PN05 + carvacrol (1.0 mg/mL) (*p* < 0.05). Phage cocktail + carvacrol (0.5 mg/mL) showed lower efficacy in inhibiting Psa growth, as the maximum bacterial density of Psa here (OD_600_ = 0.134) was significantly higher (*p* < 0.05) than it was for PN05 + carvacrol (0.5 mg/mL), and inhibition to the detection limit of the method was reached later than it was with PN05 + carvacrol (0.5 mg/mL). PN09 + carvacrol (0.5 mg/mL and 1 mg/mL, respectively) showed the lower efficiency in inhibiting Psa growth, as the maximum bacterial density of Psa (OD_600_ = 0.156 and 0.160, respectively) was significantly higher (*p* < 0.05) than it was for PN05 or phage cocktail + carvacrol (0.5 mg/mL and 1 mg/mL), and inhibition to the detection limit of the method was reached later than it was for PN05 or phage cocktail + carvacrol (0.5 mg/mL and 1 mg/mL).

**Figure 5:**
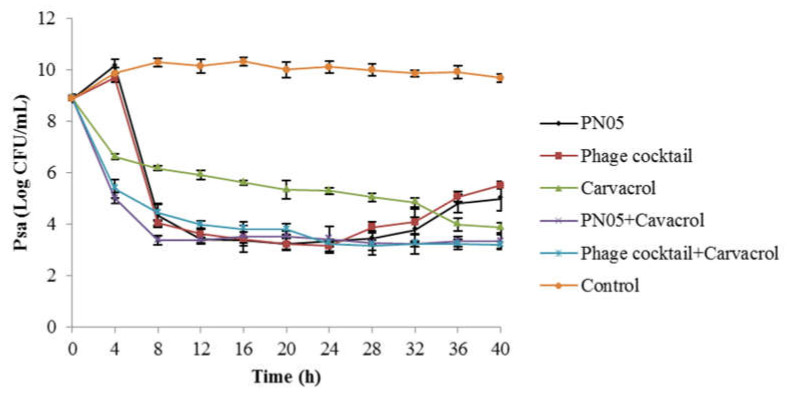
Inactivation of Psa (SCJY02-1) by phage PN05, phage cocktail and carvacrol. Data are the mean ± standard deviation of three independent assays.

According to the results of the phage inhibitory assay (Figure 4), PN05 was more effective than PN09 in inhibiting Psa (SCJY02-1) growth, as the maximum bacterial density of Psa (OD_600_ = 0.150) was significantly lower here (*p* < 0.05) than it was for PN09 alone, and inhibition to the detection limit of the method was reached earlier here than it was for PN09 alone. Therefore, PN05 was used in further inactivation assays (evaluated on the basis of colony count instead of bacterial inhibition determined, based on OD) of Psa with carvacrol (2.0 mg/mL). The results of Psa inactivation by phage and carvacrol are shown in Figure 5. After treatment with PN05, phage cocktail, carvacrol, PN05 + carvacrol and phage cocktail + carvacrol, the bacterial concentrations were reduced by 4.71, 4.19, 5.84, 6.38 and 6.52 log CFU/mL, respectively, when compared with the control treatment. After 4 h of treatment, the rate of bacterial inactivation achieved with carvacrol at 2.0 mg/mL (reduction of 3.26 log CFU/mL) was significantly higher (*p* < 0.05) than that obtained with PN05 or phage cocktail alone, when compared with the control treatment. However, after 8 h of treatment, the bacterial inactivation achieved with carvacrol at 2.0 mg/mL (reduction of 4.14 log CFU/mL) was significantly lower (*p* < 0.05) than that obtained with PN05 or phage cocktail (reduction of 5.98 and 6.26 log CFU/mL, respectively). After 24 h, Psa regrowth was observed in both the PN05 and phage cocktail treatments. When Psa was treated with PN05 or phage cocktail + carvacrol (2.0 mg/mL), the Psa concentration decreased and no regrowth was observed until the end of the experiment (40 h). The efficiency of the phage combined with carvacrol treatment significantly differed from that of carvacrol alone. For example, when compared with the control treatment, Psa inactivation by PN05 + carvacrol (reduction of 4.87 log CFU/mL) was significantly higher (*p* < 0.05) than that by carvacrol alone (reduction of 3.26 log CFU/mL) after 4 h of treatment. At 8 h, Psa inactivation by PN05 + carvacrol (reduction of 6.94 log CFU/mL) was significantly higher (*p* = 0.0065) than that by phage cocktail + carvacrol (reduction of 5.87 log CFU/mL), when compared with the control treatment.

### 3.5. Biofilm Inhibition

**Figure 6:**
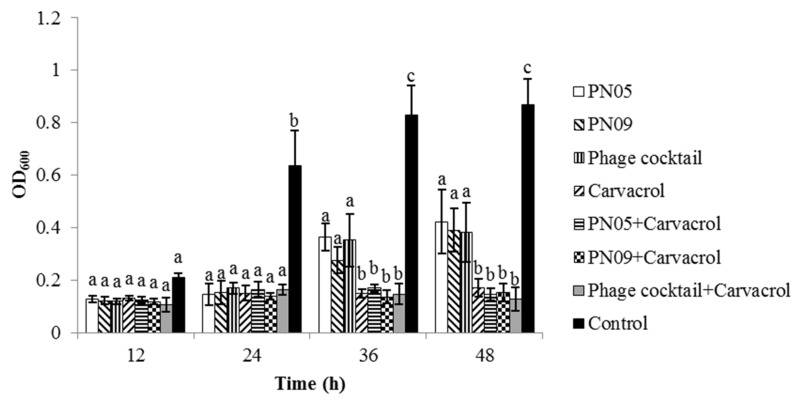
Efficacy of phages (PN05, PN09 and phage cocktail) and 2 mg/mL carvacrol in preventing Psa biofilm formation by Psa (SCJY02-1) after 12 h, 24 h, 36 h or 48 h. Different letters in the column at the same time point (12 h, 24 h, 36 h or 48 h) indicate a statistical difference (*p* < 0.05). Y-axis means the amount of biofilm formation. Data are the mean ± standard deviation of three independent assays.

The ability of the phages and carvacrol to prevent biofilm formation was investigated. Figure 6 shows that phage + carvacrol (2.0 mg/mL) and carvacrol alone (2.0 mg/mL) were effective in preventing biofilm formation for 48 h. For phage alone and phage cocktail, a significant reduction in biofilm was observed after 24 h when compared with the control group (*p* < 0.05); while the amount of biofilm started to increase from 24 h to 48 h, the amount of biofilm in the PN05, PN09 and phage cocktail treatment groups was still significantly lower than that in the control group (*p* < 0.01). At 48 h, there was no significant difference between carvacrol alone and the combination treatments. However, the amount of biofilm in the carvacrol alone group was significantly lower than that in the control group (*p* < 0.01).

### 3.6. Pre-Formed Biofilm Destruction

**Figure 7:**
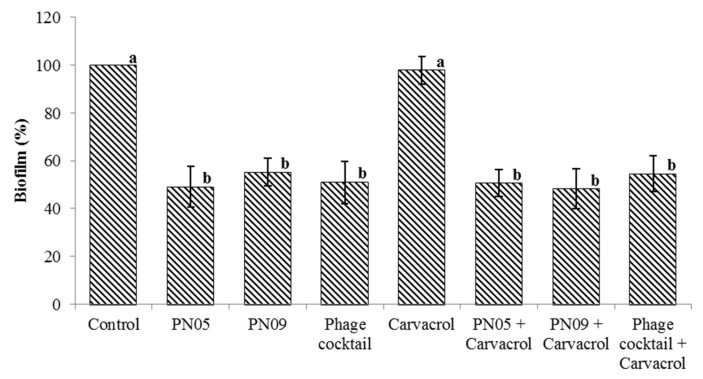
Efficacy of phages and 2.0 mg/mL carvacrol in destruction of 36-h pre-formed Psa (SCJY02-1) biofilm after 24 h. Y-axis means the percentage remaining of pre-formed biofilms. Data are the mean ± standard deviation of three independent assays. Shared letters in the column indicate no statistical difference among groups (*p* > 0.05). Different letters above bars indicate a statistically significant difference (*p* < 0.05).

Psa biofilm destruction by the phages and carvacrol is shown in Figure 7. Compared to the control wells, Psa biofilm was significantly reduced by approximately 50% after adding phage alone, phage cocktail or phage + carvacrol (2.0 mg/mL) (*p* < 0.01). Carvacrol (2.0 mg/mL) alone showed no significant effect when compared with the control treatment (*p* = 0.566).

## 4. Discussion

Psa is the major cause of kiwifruit bleeding canker and poses a severe threat to kiwifruit production worldwide [3]. Conventional Psa control agents are being faced with the emergence of resistant Psa strains, and cause environmental contamination. The application of phages in agriculture has received great attention in recent years, because phages exhibit specific infectivity toward the targeted bacterium, and have no direct negative effects on animals or plants [30,31,32]. Bae et al. used a lytic phage to control bacterial wilt in tomato [33] and Czajkowski et al. isolated phages from soil to control *Dickeya* spp., which causes potato blackleg and soft rot in potato tubers [34], resulting in the efficient inactivation of the pathogenic bacteria without affecting the plants. Previous studies have also shown that phages can effectively deactivate the *P. syringae* pathovars that are responsible for various plant diseases, including bacterial bleeding canker in kiwifruit [6,7,8,9,10]. Nevertheless, one limitation of phage therapy is that bacteria can readily develop phage resistance [35]. To avoid this, we developed a combined strategy, using phages and carvacrol, to control Psa.

We evaluated the bacteriolytic activity of PN05 and PN09 in a log-phase culture of Psa. Bacterial growth was significantly inhibited at MOIs of 0.1, 1 and 10, and was inhibited to the detection limit of the method (OD600) at MOI = 100. The results suggested that both phages were highly effective against Psa in vitro, warranting studies on their therapeutic efficacy. As shown in previous studies [36,37], the MOI is important to the efficiency of bacterial inactivation both in vitro and in vivo, and the reduction in bacterial growth increases with increasing MOI.

However, as the treatment time increased, bacterial regrowth was observed, which could be due to the emergence of phage-resistant bacteria. The emergence of phage-resistant bacterial mutants is a major concern in phage therapy [9,13,38], and this drawback has been overcome through different strategies. Mateus et al. [11], Costa et al. [14] and Hooton et al. [39] found that the emergence of phage-resistance in different bacteria can be prevented by using bacteriophage cocktails. Lopes et al. [13] reported that combined treatment with phages and antibiotics is effective in preventing the emergence of phage-resistance in *Escherichia coli*. In this study, a combination of phages and carvacrol effectively prevented the emergence of phage-resistance in Psa. Carvacrol, like other essential oils, can effectively control various bacterial pathogens, including *Escherichia coli*, *Staphylococcus aureus*, *Listeria monocytogenes* and *Pseudomonas syringae* pv. *actinidiae* [9,10,11,40]. However, to date, very few studies have evaluated the antimicrobial activity of carvacrol against Psa. We found that the MIC of carvacrol against the three tested Psa strains was 2.0 mg/mL, which was slightly higher than the value determined for other bacteria in previous studies. Moon et al. [26] reported an MIC for *S. typhimurium* of 0.5–1.0 mg/mL, and Engel et al. [10] reported an MIC against *S. aureus* of 0.66 mg/mL. In this study, the use of carvacrol at 2.0 mg/mL in the presence of the phages inhibited Psa growth, to the detection limit of the method used, for 40 h without the emergence of phage-resistant mutants. As the concentrations of carvacrol used in this study did not affect the viability of PN05 and PN09, a potential synergism between the phages and carvacrol can occur. The combination of the phage cocktail or PN05 with 2.0 mg/mL carvacrol showed a higher efficacy in inhibiting Psa and preventing the development of resistant bacteria (Figure 4c). Even at 0.5 mg/mL or 1.0 mg/mL, carvacrol improved the efficacy of the phage cocktail or PN05 in inhibiting bacterial growth when compared to phage cocktail or PN05 alone. The inhibition assay results were confirmed by the results obtained in further experiments, in which the bacterial concentration of Psa was determined by colony count. In this experiment, phage PN05 (which was the most effective against Psa in previous assays) or the phage cocktail combined with carvacrol were shown to be more effective than the treatments using PN05, phage cocktail or carvacrol alone against Psa, and no phage-resistance was observed. Together, these results show that carvacrol improves the efficacy of phages against Psa, and mitigates the development of phage-resistant mutants.

Psa can form biofilms that protect the bacterial cells from host defense and bactericidal compounds, and biofilm formation is difficult to prevent [3]. Bacteriophages may be used to avoid biofilm formation, or even to destroy established biofilm. We found that the single phage suspensions and phage cocktail were effective in inhibiting biofilm formation for 24 h, after which biofilm regrowth was observed. However, carvacrol alone and carvacrol combined with the phages effectively prevented Psa biofilm formation for 48 h. Biofilm regrowth in phage treatments might have resulted from the emergence of phage-resistant mutants. Similarly, other studies have reported that phages alone, as well as carvacrol alone, can inhibit biofilm formation in *Cronobacter sakazakii* and *Enterobacter cloacae* [29,41]. However, Burt et al. [16] found that carvacrol had no effect on the biofilm formation of *P. aeruginosa*. In this study, the phages and phages combined with carvacrol at 2.0 mg/mL were able to destroy pre-formed biofilm, whereas carvacrol at 2.0 mg/mL had no significant effect. These findings indicated that PN05 and PN09, but not carvacrol at 2.0 mg/mL, were effective in removing existing biofilm. These results were consistent with those obtained by Gu et al. [42] and Burt et al. [24], who found that phages were able to reduce the pre-formed biofilm of uropathogenic *Escherichia coli*, whereas carvacrol had little activity against the pre-formed biofilms of *Chromobacterium violaceum*, *S. typhimurium*, and *S. aureus*. These results demonstrated that combining phages with carvacrol at 2.0 mg/mL is a potential method to control Psa biofilm.

## 5. Conclusions

The combined treatment, with phages and carvacrol at 2.0 mg/mL, effectively reduced the Psa concentration, preventing the emergence of phage-resistant mutants. Phages combined with carvacrol can not only effectively control biofilm development, but also destroy pre-formed Psa biofilms. Consequently, phage therapy may be a potential method for controlling Psa in the kiwifruit industry, and combination with the natural antimicrobial carvacrol can increase phage efficacy in controlling Psa.
